# Improvements in spatiotemporal outcomes, but not in recruitment of automatic postural responses, are correlated with improved step quality following perturbation-based balance training in chronic stroke

**DOI:** 10.3389/fspor.2022.1008236

**Published:** 2022-11-17

**Authors:** Wouter H. A. Staring, Hanneke J. R. van Duijnhoven, Jolanda M. B. Roelofs, Sarah Zandvliet, Jasper den Boer, Frits C. Lem, Alexander C. H. Geurts, Vivian Weerdesteyn

**Affiliations:** ^1^Department of Rehabilitation, Donders Institute for Brain, Cognition and Behaviour, Radboud University Medical Center, Nijmegen, Netherlands; ^2^Department of Rehabilitation, Sint Maartenskliniek, Nijmegen, Netherlands

**Keywords:** perturbation-based balance training, balance recovery, stroke, reactive stepping, spatiotemporal outcomes, step quality

## Abstract

**Introduction:**

People with stroke often exhibit balance impairments, even in the chronic phase. Perturbation-based balance training (PBT) is a therapy that has yielded promising results in healthy elderly and several patient populations. Here, we present a threefold approach showing changes in people with chronic stroke after PBT on the level of recruitment of automatic postural responses (APR), step parameters and step quality. In addition, we provide insight into possible correlations across these outcomes and their changes after PBT.

**Methods:**

We performed a complementary analysis of a recent PBT study. Participants received a 5-week PBT on the Radboud Fall simulator. During pre- and post-intervention assessments participants were exposed to platform translations in forward and backward directions. We performed electromyography of lower leg muscles to identify changes in APR recruitment. In addition, 3D kinematic data of stepping behavior was collected. We determined pre-post changes in muscle onset, magnitude and modulation of recruitment, step characteristics, and step quality. Subsequently, we determined whether improvements in step or muscle characteristics were correlated with improved step quality.

**Results:**

We observed a faster gastrocnemius muscle onset in the stance and stepping leg during backward stepping. During forward stepping we found a trend toward a faster tibialis anterior muscle onset in the stepping leg. We observed no changes in modulation or magnitude of muscle recruitment. Leg angles improved by 2.3° in forward stepping and 2.5° in backward stepping. The improvement in leg angle during forward stepping was accompanied by a −4.1°change in trunk angle, indicating a more upright position. Step length, duration and velocity improved in both directions. Changes in spatiotemporal characteristics were strongly correlated with improvements in leg angle, but no significant correlations were observed of muscle onset or recruitment with leg or trunk angle.

**Conclusion:**

PBT leads to a multi-factorial improvement in onset of APR, spatiotemporal characteristics of stepping, and reactive step quality in people with chronic stroke. However, current changes in APR onset were not correlated with improvement in step quality. Therefore, we suggest that, in addition to spatiotemporal outcomes, other characteristics of muscle recruitment or behavioral substitution may induce step quality improvement after PBT.

## Introduction

People with stroke (PwS) often exhibit balance impairments, even in the chronic phase (1). These balance impairments have a detrimental effect on their mobility and daily life independence (2) and contribute to the high fall risk post stroke (3). Specifically, a stroke results in an at least two-fold increased risk of falls (4), which may have severe consequences such as hip fractures on the paretic side (5). While exercise interventions with a balance component may be effective in reducing fall risk after stroke (6), it remains unclear which type of balance training is most effective. For the development of more effective falls prevention programs, it is crucial to understand how stroke-related balance deficits respond to training.

One promising training target is the capacity to recover from balance perturbations. The main rationale for focusing on balance-recovery responses to help prevent an actual fall lies in their utility in a wide variety of situations that may induce a loss of balance (e.g., misstep, trip or collision with another pedestrian). In PwS, this so-called reactive balance capacity is often impaired (7), with stepping responses showing more profound deficits than feet-in-place balance recovery strategies. Stroke-related deficits have been observed in different aspects of reactive stepping. First, PwS exhibit deficient automatic postural responses (APRs). APRs are fast automatic muscle responses evoked by balance perturbations and act as a first line of defense to counteract loss of balance (8). These deficiencies are evident by delayed muscle onsets (9) and reduced amplitudes of APRs (10), as well as poorer muscle coordination patterns during the APR time window (11). Second, a stroke affects the spatiotemporal characteristics of reactive stepping. Specifically, PwS may demonstrate a delayed step onset (12, 13) and a smaller step length (14) compared to healthy individuals. Lastly, balance recovery steps in PwS are less effective in ‘catching' the falling center of mass (CoM), as shown by outcome measures that capture the relationship between CoM and the base of support at the instance of foot contact (9, 15).

Recent evidence suggests that training involving repeated exposure to balance perturbations (perturbation-based balance training; PBT) can improve stroke-related deficits in reactive stepping (16, 17) and may reduce fall risk (18). At the level of APRs, faster muscle responses were found after PBT in healthy individuals (19), but the effect of PBT on the size and direction-dependent amplitude modulation of the APRs have not yet been studied. At the level of spatiotemporal step characteristics, PBT has shown to improve step length (20), but its effect on other variables such as step velocity or step time remains unclear. Importantly, studies also show that reactive stepping performance improved following PBT (21, 22). Specifically, after PBT, PwS required fewer steps and exhibited a more favorable body configuration at stepping foot contact, as evidenced by a larger leg angle (stepping foot placed further ahead of the pelvis in the direction of perturbation) (16).

Still, limited evidence is available on the effects of PBT at the level of APRs, spatiotemporal variables and stepping performance in PwS. Moreover, it is unclear whether improvements in one respect would translate to the other. As such, specific insights into the mechanisms underlying improvement of reactive stepping performance through PBT in PwS is limited. To obtain more insight in these mechanisms, we performed additional analyses on the data collected by van Duijnhoven et al. (16), who previously reported the beneficial effects of PBT on stepping leg angles following perturbations induced by a moveable platform. The aim of our study was threefold. Our first aim was to provide a comprehensive characterization of PBT-induced changes in APRs and spatiotemporal characteristics of reactive stepping in PwS. At the level of APRs we were interested in onset latencies, response amplitudes and direction-dependent modulation of activity of the prime movers (in the forward and backward direction). Spatiotemporal variables involved step onset, step duration, step velocity and step length. Our second aim was to determine whether step quality in response to forward and backward perturbations would improve in terms of trunk angle at foot contact, in addition to the previously reported improvements in leg angle. Our third aim was to determine which improvements in APR and spatiotemporal variables would underlie improvements in step quality, as quantified by the body configuration at foot contact (i.e., leg and trunk angles).

## Methods

### Participants

As described in van Duijnhoven et al., 20 participants in the chronic phase (> 6 months) of stroke were recruited from Nijmegen and the surrounding area. Detailed patient characteristics can be found in Table 1. Participants had to be able to stand and walk independently (Functional Ambulation Categories >3). They were excluded if (1) they had other neurological or musculoskeletal conditions affecting balance, (2) used drugs affecting balance, (3) had severe cognitive problems (Mini Mental State Examination < 24) or persistent unilateral spatial neglect (Star Cancellation Test < 44), or (4) had behavioral problems interfering with compliance to the protocol. For the current study we only selected those participants of whom data was collected at pre- and post- training assessments and who stepped with the same leg during both assessments (*N* = 18). The study protocol was approved by the Medical Ethical Board of the region Arnhem-Nijmegen and all participants gave written informed consent in accordance with the Declaration of Helsinki.

**Table 1:** Participant characteristics (*n* = 18).

Sex (m/f)	10/8
Age (years)	59 (8.3)
Months since stroke	54 (40)
Stroke type (Ischemic / hemmorhagic)	12/6
Affected body side (left /right)	11/7
MMSE	27,8 (2)
MI-LE (range 0–100%)	64 (19)
ABC-6 Scale (range 0–100%)	42 (25)
Fall history (number of falls in previous year)	1.4 (1.8)
FMA-LE (range 0–28)	20 (5)
FAC (4/5)	4/14

MMSE, Mini Mental State Examination; MI-LE, Motricity Index lower extremity; ABC-6 Scale, Activities-specific Balance Confidence 6 Scale, FMA-LE, Fugl-Meyer Assessment lower extremity score without coordination domain; FAC, Functional Ambulation Categories. Values are presented in means (SD).

### Study design and intervention

Participants received a 5-week PBT on the Radboud Falls Simulator, with a total of 10 sessions. The Radboud Falls Simulator is a movable platform that can induce balance perturbations by horizontal translations in multiple directions (see Supplementary Videos). Participants received 45 min of training, twice a week. Training difficulty was increased during each training session in a participant-specific manner, more specifically, through increasing the intensity of the perturbation, the unpredictability of perturbation direction, or by adding secondary (cognitive) tasks (dual-tasking). Detailed information on the study design can be found in van Duijnhoven et al. (16) and the training protocol can be accessed directly *via* this link, https://www.frontiersin.org/files/Articles/410755/fneur-09-00980-HTML/image_m/fneur-09-00980-t002.jpg.

### Experimental procedure

During pre- and post-training assessments participants were exposed to unpredictable translational platform perturbations that consisted of an acceleration phase (300 ms), a constant velocity phase (500 ms), and a deceleration phase (300 ms) (23) initiated after a random delay. Participants stood on the platform with their preferred shoes and wore a safety harness. They were instructed to recover their balance with one single step. To standardize the perturbation difficulty across individuals with different balance recovery capacities, a participant-specific perturbation intensity was determined during the pre-training assessment. To this end, the multiple stepping threshold (MST) was determined by gradually increasing the perturbation intensity (acceleration) in each direction. The perturbation direction refers to the direction of stepping, such that a forward perturbation would induce a forward step (i.e., platform moving backwards). The direction-specific MST was defined as the maximum intensity at which a participant was able to recover his/her balance with one step. To allow comparison of stepping characteristics between the pre- and post-training, trials at the same intensity (MST and MST+0.125 m/s^2^) were collected during both assessments. Within this respective study the average MST value for forward perturbations was 3.03 m/s^2^ (SD = 1.4 m/s^2^) and for backward perturbations 2.2 m/s^2^ (SD = 0.88 m/s^2^).

### Data collection

Reactive stepping responses were recorded at 100 Hz using an 8-camera 3D motion capture system (Vicon Motion Systems, Oxford, UK). Reflective markers were placed on anatomical landmarks according to the Plug-in-Gait full body model. In addition, we recorded bilateral electromyography (EMG) from Rectus Femoris (RF), Tibialis Anterior (TA), and Gastrocnemius Medialis (GM). EMG electrodes were placed according to SENIAM guidelines (24) and recorded at 1,000 Hz (ZeroWire by Aurion, Italy).

### Data processing

Marker data were low-pass filtered at 5 Hz (2^nd^ order Butterworth). Subsequently marker data and EMG data were processed by a custom-written MATLAB (MATLAB2018a) script. Raw EMG was bandpass filtered between 10 and 450 Hz, rectified and low-pass filtered at 40 Hz. Rectified EMG was averaged across trials with similar platform direction. Subsequent analysis was performed on the averaged rectified EMG.

### Data analysis

The onset of muscle activity was determined for the prime movers during the APR for each perturbation direction (GM for forward perturbations and TA and RF for backward perturbations). Onset latencies were determined by means of a semi-automatic algorithm that selected the instant at which the averaged rectified EMG exceeded a threshold of 2 SDs above the mean of pre-perturbation activity (500 ms) (11). Perturbation onset was determined through a synchronization trigger sent from the platform. Visual inspection of the trials was performed to verify the accuracy of the onset.

To assess muscle recruitment and modulation during the APR we calculated the integrated rectified EMG (iEMG) and the Modulation Index during the first 75 ms after the first muscle onset in a given direction. Thus, during forward stepping, iEMG was calculated post GM onset and, vice versa, during backward stepping post TA onset.

As described by the studies of Lang et al. (25) and Kelly et al. (26) the Modulation Index can be used to quantify relative changes in activity when a muscle would serve as an agonist compared to when it would be an antagonist. In addition, it can serve as a measure to express a persons' ability to scale task-specific muscular recruitment. As for the APR, the GM acts as an agonist during forward perturbations and as an antagonist during backward perturbations, whereas the opposite is true for the TA. The Modulation Index was defined as described by EQ1.


(1) $$\begin{array}{l} Modulation\;Index=100^{*}\frac{EMG_{75ms}^{ag}\;{-\;EMG}_{75ms}^{ant}}{EMG_{75ms}^{ag}}. \end{array}$$

$EMG_{75ms}^{ag}$ is the mean EMG within the 75 ms window of muscle in the agonistic direction and $EMG_{75ms}^{ant}$ is the mean EMG within the 75 ms window in the antagonistic direction. A higher MI indicates a greater recruitment of the muscle in its expected behavior.

Spatiotemporal stepping behavior was assessed by determining step onset, step length, step duration and step velocity. Step onset was defined by marker data as the moment at which the vertical velocity component of the heel or toe marker surpassed a threshold of 0.2 m/s. To calculate the subsequent spatiotemporal stepping behavior parameters, foot contact was defined as the moment when the vertical velocity component of the heel or toe went below 0.2 m/s after step onset. Step length was then calculated as the distance covered by the toe marker from step onset to foot contact. Step duration was defined as the duration of step onset until foot contact, and step velocity was calculated as step length divided by step duration.

**Figure 1:**
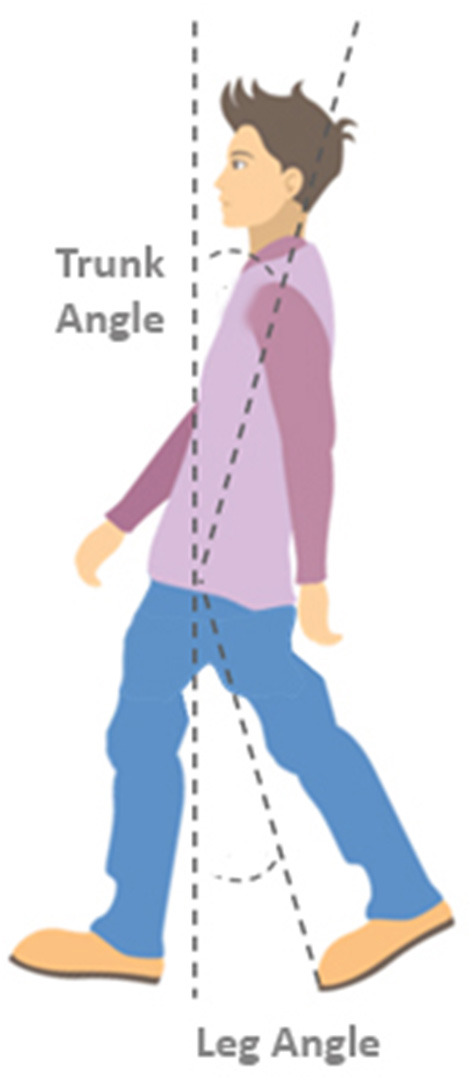
Definitions of leg and trunk angles. The trunk angle is defined as the angle between the vertical and a line connecting the mid-shoulder to the mid-pelvis position at the instant of stepping foot contact. The leg angle is defined as the angle between the vertical and a line connecting the mid-pelvis and the 2^nd^ metatarsal of the stepping foot.

To characterize step quality, body configuration at first stepping foot contact was determined. Body configuration outcomes included the vertical inclination angle of the leg segment (defined as the angle between the vertical and a line connecting the mid-pelvis and the second metatarsal of the stepping leg; see Figure 1), i.e., the leg angle, and the trunk segment (defined as the angle between the vertical and a line connecting the mid-pelvis and mid-shoulder position), i.e., the trunk angle.

### Statistical analysis

Since outcome measures did not significantly differ between the two perturbation intensities (MST and MST+0.125 m/s^2^), subsequent comparisons were performed on the two intensities collectively. To evaluate between session differences (Δ_pre − post)_ we performed two-tailed paired *t-*tests on the averaged outcomes. Additionally, we identified potential determinants of improvements in body configuration (leg and trunk angle) through a similar procedure described by de Kam et al. (9, 15). First, we selected those spatiotemporal and muscular outcome measures that improved significantly after training. Subsequently, from those outcome measures Pearson's correlations coefficient were used to identify which outcome measures were significantly correlated (*p* < 0.05) with changes in body configuration. Variables that were significantly correlated in these univariate analyses into multivariate forward step-wise linear regression models with changes in leg and trunk angle as dependent variables for each platform direction. Statistical analyses were performed with SPSS (version 25.0). P < 0.05 was considered statistically significant.

## Results

### Amplitude and modulation of automatic postural responses

In the post-training assessment, we observed faster automatic postural responses compared to pre-training, while APR amplitude and activity modulation did not change. Specifically, during forward stepping we observed a small but significant shortening in the gastrocnemius onset latency in the stance and stepping leg (*stepping leg:* Δ_pre − post_ *5 ms* ± 7*, p* = *0.01; stance leg:* Δ_pre − post_ *5 ms* ± 7*, p* = *0.02)*. For backward stepping, we observed a trend toward a faster tibialis anterior onset in the stepping leg (Δ_pre − post_ *4.4ms* ± 9.2*, p* = *0.06)* but not in the stance leg (*p* = 0.16). Onset latencies of the rectus femoris did not change in response to training (*stepping leg p* = 0.20, *stance leg p* = 0.68).

### Step quality and spatiotemporal characteristics

**Figure 2:**
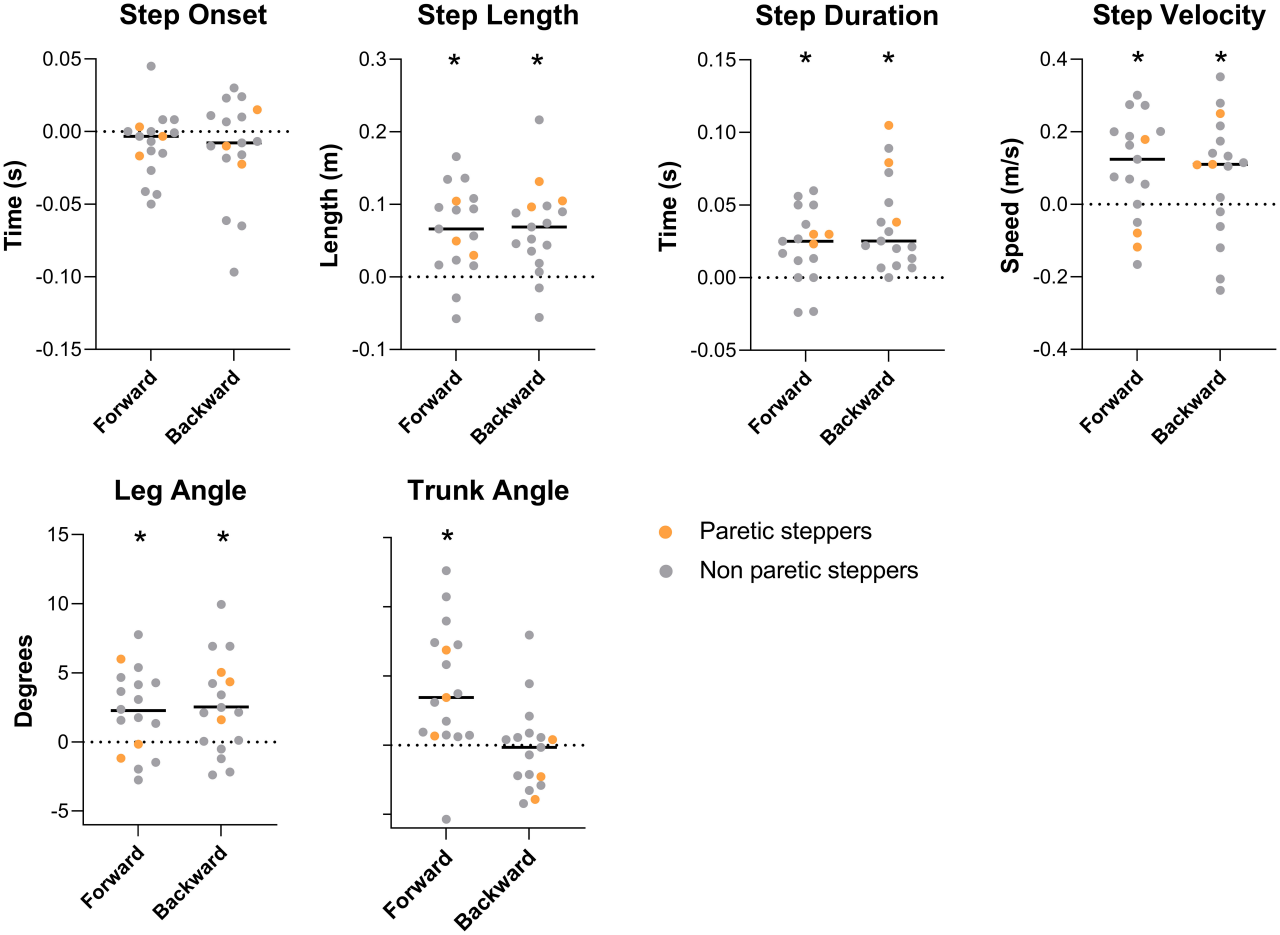
Spatiotemporal and body configurational outcome effect of training. Outcome measures are expressed as pre-post training differences. Bars represent group average, dots represent individual data points; participants who stepped with the paretic leg are indicated as orange dots. * Represents a significant pre-post training difference (*P* < 0.05).

As previously reported by van Duijnhoven et al. (16), participants demonstrated improved reactive step quality following the training intervention. The leg angles increased for both the forward and backward perturbations [forward (fwd): Δ_pre − post_ = 2.3 ± 3.0°, *p* < 0.01; backward (bwd): Δ_pre − post_ = 2.5° ± 3.5°, *p* < 0.01; Figure 2], indicating that the stepping foot was placed further ahead of the pelvis. Complementary to our previous study, we found and improvement in trunk angle for forward stepping following training. Specifically, the trunk angle decreased, which indicates a more vertically oriented trunk (Δ_pre − post_ = −4.1 ± 1.1°, *p* < 0.01; Figure 2). On the other hand, no changes were observed in trunk angles after backward perturbations (Table 2).

**Table 2:** Mean (SD) pre and post-training values of step quality and step characteristics.

	**Pre intervention**	**Post intervention**	***P*-value**
**Forward stepping**			
Leg angle (°)	21.1 (3.4)	23.4 (2.9)	**0.007**
Trunk angle (°)	22.3 (7.8)	18.1 (7.3)	**0.002**
Step onset (s)	0.29 (0.04)	0.28 (0.04)	0.117
Step length (m)	0.48 (0.2)	0.55 (0.1)	**< 0.001**
Step duration (s)	0.3 (0.04)	0.33 (0.04)	**0.002**
Step velocity (m/s)	1.56 (0.4)	1.66 (0.4)	**0.011**
**Backward stepping**			
Leg angle (°)	−1.6 (6.6)	0.9 (5.6)	**0.008**
Trunk angle (°)	2.1 (7.2)	2.3 (7)	0.55
Step onset (s)	0.32 (0.05)	0.32 (0.04)	0.19
Step length (m)	0.37 (0.2)	0.44 (0.2)	**0.001**
Step duration (s)	0.25 (0.05)	0.29 (0.04)	**< 0.001**
Step velocity (m/s)	1.4 (0.5)	1.47 (0.4)	*0.06*

Statistically significant different outcome measures are highlighted in bold *P* < 0.05.

The observed improvements in step quality were accompanied by changes in some, but not all, spatiotemporal step characteristics. Specifically, greater step lengths were observed in the post vs. pre-training assessments for both forward and backward perturbations (fwd: Δ_pre − post_ = 0.06 ± 0.06 m, *p* < 0.01; bwd: Δ_pre − post_ = 0.06 ± 0.06 m, *p* < 0.01);. Similarly, step durations increased for both perturbation directions (fwd: Δ_pre − post_ = 0.02 ± 0.02 s, *p* < 0.01; bwd: Δ_pre − post_ = 0.03 ± 0.03 s, *p* < 0.01). An increased step velocity was observed for forward perturbations (Δ_pre − post_ = 0.09 ± 0.14 m/s, *p* = 0.01), with a similar trend for backward steps (Δ_pre − post_ = 0.07 ± 0.16 m/s, *p* = 0.06). Step onsets were not different between the pre- and post-training assessments.

### Strong correlations between leg angle and spatiotemporal outcomes

The univariate analyses yielded moderate to strong correlations of leg angle with step length (r = 0.87, *p* < 0.01), step duration (r = 0.52, *p* < 0.01), and step velocity (r = 0.91, *p* < 0.01) for forward stepping. For backward stepping moderate to strong correlations of leg angle were observed with step length (r = 0.87, *p* < 0.01), step duration (r = 0.64, *p* < 0.01) and step velocity (r = 0.67, *p* < 0.01). None of the outcomes in muscle recruitment were correlated with changes in leg angle. The subsequent multivariate step-wise regression analyses for leg angle revealed that during forward perturbations, an increase in step velocity and step length together explained 90% of the variance in the pre-post leg angle differences (F = 62.7 R^2^ = 0.90, *p* < 0.001, β^velocity^ = 12.01, *p* < 0.01, β^length^ = 22.00, *p* < 0.01). For backward stepping, an increase in step length explained 76% of the variance in pre-post leg angle differences (F = 47.57, R^2^ = 0.76, *p* < 0.001 β = 48.77). None of the outcome measures used in the current study were significantly correlated to changes in trunk angle for either backward or forward perturbations.

## Discussion

We aimed to evaluate whether PBT would improve APRs and spatiotemporal characteristics of reactive stepping in people in the chronic phase of stroke. In addition, we determined whether improvements in leg and trunk angles could be induced by underlying spatiotemporal and neuromuscular components. At the level of APRs, our findings showed a slight shortening in onset latencies during forward perturbations, with a similar tendency in the stepping leg during backward perturbations, whereas no differences were observed in magnitude or modulation. Analysis of spatiotemporal characteristics revealed improvements in step length, step duration, and step velocity. Leg angles improved after PBT in both perturbation directions, whereas trunk angles only significantly improved in forward steps. Improvements in leg angle were largely explained by larger step lengths and faster step velocities after training, whereas improvements in APR latencies did not contribute significantly to the changes in leg angle. None of the spatiotemporal or EMG parameters correlated with improvements in trunk angle.

The presently reported changes in trunk angle at step contact following forward perturbations complement the previously reported improvements in leg angle (16). Specifically, we observed a more upright trunk position at the post-training assessment, which is in accordance with the effects of trip-specific training as reported by Pigman et al. (20) and Nevisipour et al. (27). Smaller forward trunk rotation angles or angular velocities are correlated with better postural stability (28, 29). The more upright position after training indicates that participants were able to generate a more effective response to counteract the induced forward angular momentum. This finding, in combination with the larger leg angles, demonstrates that our participants had improved their reactive stepping performance following PBT, which is in agreement with observations from other PBT studies in people with stroke (22, 30). Yet, despite the growing evidence of beneficial effects of PBT on performance-related outcomes, there is a lack of insight into the mechanisms underlying these improvements. Therefore, we performed a comprehensive analysis of possible determinants that may have contributed to the observed improvements in reactive step quality.

We investigated training-induced changes in APR recruitment, because PwS often have persistent deficits in APR onset latency, magnitude and coordination patterns, mainly on the paretic side (9–11). The vast majority of our participants used their paretic leg as the stance leg (*n* = 14), and Table 3 shows that APR recruitment commenced ~10–20 ms later with lower magnitudes and poorer modulation in the stance compared to the stepping leg. These observations suggest that participants who presented with APR deficits could potentially improve by training. Indeed, after PBT we observed faster APR onsets in lower-leg muscles, which finding complements the very sparse studies that have reported effects of training on APRs in PwS (31, 32), with only a single study thus far showing significant improvements in the paretic leg (33). The average shortening of onset latencies, however, was rather modest (5–6 ms), which limits the potential clinical relevance.

**Table 3:** Mean (SD) pre and post-training values of muscle onset latencies, amplitudes and modulation indices.

	**Pre intervention**	**Post intervention**	***P*-value**
**Stepping direction**			
***Forward***			
Stepping leg			
*Gm Onset (ms)*	157 (16)	152 (15)	0.01*
*Gm iEmg (μV)*	11.2 (10.1)	11.0 (5.8)	0.9
Stance leg			
*Gm Onset (ms)*	174 (23)	168 (23)	0.02*
*Gm iEmg (μV)*	5.9 (3.8)	6.4 (3.9)	0.55
***Backward***			
Stepping leg			
*Ta Onset (ms)*	153 (17)	149 (17)	***0.06***
*Rf Onset (ms)*	169 (22)	165 (22)	0.16
*Ta iEmg (μV)*	17.0 (7.4)	15.6 (10.6)	0.33
*Rf iEMg (μV)*	2.7 (2.6)	2.9 (2.4)	0.66
Stance leg			
*Ta Onset (ms)*	163 (17)	162 (19)	0.68
*Rf Onset (ms)*	187 (19)	184 (18)	0.21
*Ta iEmg (μV)*	11.5 (5.7)	10 (6.7)	0.52
*Rf iEMg (μV)*	3.2 (4.2)	2.6 (2.4)	0.49
**Modulation index (%)**			
Stepping leg			
*Ta*	82 (15)	80 (31)	0.83
*Rf*	57 (26)	58 (32)	0.73
*Gm*	81 (10)	84 (11)	0.37
Stance leg			
*Ta*	70 (36)	78 (23)	0.43
*Rf*	20 (104)	40 (35)	0.10
*Gm*	60 (45)	65 (47)	0.24

Statistically significant different outcome measures are highlighted in bold *P* < 0.05.

In accordance with studies indicating direction-specific deficits in coordination of APRs in the paretic leg of people with chronic stroke (9, 34), the modulation index was generally lower (i.e., poorer) in the (predominantly paretic) stance leg of our participants. Indeed, the mean pre-intervention modulation indices in the stance leg of 20–70% are all below the average of 71% as reported for a combined group of healthy older individuals and people with Parkinson's disease (26). This result shows that there was room for improvement in stance-leg APR recruitment in our participants, yet we did not observe PBT-related gains in the magnitude of agonistic muscle recruitment (iEMG), nor in direction-dependent modulation (Modulation Index). The present lack of chance in APR recruitment is reminiscent of previous work on recovery of gait in the subacute phase after stroke (35, 36). These studies showed that walking ability substantially improved in the absence of significant changes in aberrant muscle coordination, presumably through behavioral substitution rather than restoration of function. Likewise, we surmise that the beneficial effects of our PBT intervention on reactive step quality, in the absence of improvements in APR recruitment, may also point at behavioral substitution rather than restoration of function (37, 38).

Following the PBT intervention, we found significant improvements in spatiotemporal outcomes of reactive stepping, with greater step lengths, step duration and step velocity being observed in both perturbation directions. These findings complement previous studies that reported variable effects of PBT on one or more spatiotemporal step characteristics, in addition to consistently positive effects of PBT on reactive stepping performance (17, 20, 34). Our regression analyses provided novel insight into the relationships between changes in spatiotemporal step characteristics on the one hand, and gains in reactive step quality on the other hand. Increased step lengths were significantly associated with larger leg angles (i.e., better step quality) in both backward and forward perturbation directions, whereas increased step velocity was identified as an additional significant contributor in the forward direction only. Under the assumption of unchanged perturbation-induced CoM dynamics, a larger step length provides a greater lever arm for the ground reaction force to produce torques that counteract the CoM movement. A faster stepping velocity, in turn, reduces the time for the CoM to accelerate and displace relative to the base of support, thus making it easier to be “caught” at foot strike. Hence, these improvements in spatiotemporal step characteristics provide a biomechanically plausible explanation for the observed gains in reactive step quality.

While in our regression models, faster APR onset latencies did not additionally contribute to the explained variance in step quality improvements, it cannot be excluded that a faster response in the support leg (paretic leg for the majority of participants) may have been beneficial. The faster response could reduce the angular momentum generated by the perturbation, thereby providing extra time for the stepping leg and allowing better clearance for proper positioning of the stepping limb (39). Yet as stated before, the modest 5–6 ms shortening that we observed in the present study may not represent a substantial benefit.

A limitation of our study is that our analysis did not permit comparing the effects of training on the paretic and non-paretic legs separately. We allowed our participants to self-select their stepping limb as this would resemble their most instinctive stepping response, but this instruction resulted in few participants selecting their paretic leg. Therefore, we suggest for future studies to also examine imposed stepping with the paretic and non-paretic leg separately. In addition, to improve the generalizability of the current results toward a greater population of PwS, it would be preferable to increase the sample size.

In summary, the current improvements in step quality after PBT in our group of participants people with chronic stroke were largely explained by improved spatiotemporal characteristics and not by changes in APR recruitment. For gaining further insight into the observed effects of training on spatiotemporal characteristics of reactive stepping, it may be of interest to study changes in muscle recruitment during execution of the recovery step itself, in addition to the present focus on the APR that precedes stepping.

## Data availability statement

The original contributions presented in the study are included in the article/Supplementary material, further inquiries can be directed to the corresponding author.

## Ethics statement

The studies involving human participants were reviewed and approved by the Medical Ethical Board of the region Arnhem-Nijmegen. The patients/participants provided their written informed consent to participate in this study.

## Author contributions

WS: analyzed data and wrote paper. HD and JR: designed, conducted experiment, and critical revision. SZ: supervision throughout analysis and draft of the manuscript. JB and FL: eligibility assessment of participants and critical revision. AG: eligibility assessment of participants, supervised WS throughout the study, and provided feedback. VW: designed experiment, supervised WS throughout the study, and provided feedback. All authors contributed to the article and approved the submitted version.

## Funding

WS is funded by a Netherlands Organization for Scientific Research (NWO). VIDI grant awarded to VW (No. 9171736), Project- Roads to recovery.

## Conflict of interest

Authors FL, AG, and VW were employed by Sint Maartenskliniek. The remaining authors declare that the research was conducted in the absence of any commercial or financial relationships that could be construed as a potential conflict of interest.

## Publisher's note

All claims expressed in this article are solely those of the authors and do not necessarily represent those of their affiliated organizations, or those of the publisher, the editors and the reviewers. Any product that may be evaluated in this article, or claim that may be made by its manufacturer, is not guaranteed or endorsed by the publisher.

## References

[1] Weerdesteyn V de Niet M van Duijnhoven HJ Geurts AC. Falls in individuals with stroke. J Rehabil Res Dev. (2008) 45:1195–213. 10.1682/JRRD.2007.09.0145 19235120

[2] Schmid AA Van Puymbroeck M Altenburger PA Miller KK Combs SA Page SJ. Balance is associated with quality of life in chronic stroke. Top Stroke Rehabil. (2013) 20:340–6. 10.1310/tsr2004-340 23893833

[3] Batchelor FA Mackintosh SF Said CM Hill KD. Falls after stroke. Int J Stroke. (2012) 7:482–90. 10.1111/j.1747-4949.2012.00796.x 22494388

[4] Simpson LA Miller WC Eng JJ. Effect of stroke on fall rate, location and predictors: a prospective comparison of older adults with and without stroke. PLoS ONE. (2011) 6:e19431. 10.1371/journal.pone.0019431 21559367 PMC3084849

[5] Pouwels S Lalmohamed A Leufkens B de Boer A Cooper C van Staa T. Risk of hip/femur fracture after stroke: a population-based case-control study. Stroke. (2009) 40:3281–5. 10.1161/STROKEAHA.109.554055 19661475

[6] Denissen S Staring W Kunkel D Pickering RM Lennon S Geurts AC. Interventions for preventing falls in people after stroke. Cochrane Database Syst Rev. (2019) 10:CD008728. 10.1002/14651858.CD008728.pub3 31573069 PMC6770464

[7] Mansfield A Inness EL Wong JS Fraser JE McIlroy WE. Is impaired control of reactive stepping related to falls during inpatient stroke rehabilitation? Neurorehabil Neural Repair. (2013) 27:526–33. 10.1177/1545968313478486 23504551

[8] Moore SP Rushmer DS Windus SL Nashner LM. Human automatic postural responses: responses to horizontal perturbations of stance in multiple directions. Exp Brain Res. (1988) 73:648–58. 10.1007/BF00406624 3224674

[9] de Kam D Roelofs JMB Bruijnes A Geurts ACH Weerdesteyn V. The next step in understanding impaired reactive balance control in people with stroke: the role of defective early automatic postural responses. Neurorehabil Neural Repair. (2017) 31:708–16. 10.1177/1545968317718267 28691582 PMC5714159

[10] Marigold DS Eng JJ. Altered timing of postural reflexes contributes to falling in persons with chronic stroke. Exp Brain Res. (2006) 171:459–68. 10.1007/s00221-005-0293-6 16418855 PMC3226801

[11] de Kam D Geurts AC Weerdesteyn V Torres-Oviedo G. Direction-specific instability poststroke is associated with deficient motor modules for balance control. Neurorehabil Neural Repair. (2018) 32:655–66. 10.1177/1545968318783884 29954244

[12] Martinez KM Rogers MW Blackinton MT Cheng MS Mille ML. Perturbation-induced stepping post-stroke: a pilot study demonstrating altered strategies of both legs. Front Neurol. (2019) 10:711. 10.3389/fneur.2019.00711 31333566 PMC6618516

[13] Gray VL Yang CL Fujimoto M McCombe Waller S Rogers MW. Stepping characteristics during externally induced lateral reactive and voluntary steps in chronic stroke. Gait Posture. (2019) 71:198–204. 10.1016/j.gaitpost.2019.05.001 31078009 PMC6589388

[14] Salot P Patel P Bhatt T. Reactive balance in individuals with chronic stroke: biomechanical factors related to perturbation-induced backward falling. Phys Ther. (2016) 96:338–47. 10.2522/ptj.20150197 26206220

[15] de Kam D Roelofs JMB Geurts ACH Weerdesteyn V. Body configuration at first stepping-foot contact predicts backward balance recovery capacity in people with chronic stroke. PLoS ONE. (2018) 13:e0192961. 10.1371/journal.pone.0192961 29470535 PMC5823379

[16] van Duijnhoven HJR Roelofs JMB den Boer JJ Lem FC Hofman R van Bon GEA. Perturbation-based balance training to improve step quality in the chronic phase after stroke: a proof-of-concept study. Front Neurol. (2018) 9:980. 10.3389/fneur.2018.00980 30524360 PMC6261972

[17] Schinkel-Ivy A Huntley AH Aqui A Mansfield A. Does perturbation-based balance training improve control of reactive stepping in individuals with chronic stroke? J Stroke Cerebrovasc Dis. (2019) 28:935–43. 10.1016/j.jstrokecerebrovasdis.2018.12.011 30630753

[18] Mansfield A Aqui A Danells CJ Knorr S Centen A DePaul VG. Does perturbation-based balance training prevent falls among individuals with chronic stroke? A randomised controlled trial. BMJ Open. (2018) 8:e021510. 10.1136/bmjopen-2018-021510 30121600 PMC6104758

[19] Krause A Freyler K Gollhofer A Stocker T Bruderlin U Colin R. Neuromuscular and kinematic adaptation in response to reactive balance training - a randomized controlled study regarding fall prevention. Front Physiol. (2018) 9:1075. 10.3389/fphys.2018.01075 30131722 PMC6090079

[20] Pigman J Reisman DS Pohlig RT Jeka JJ Wright TR Conner BC. Anterior fall-recovery training applied to individuals with chronic stroke. Clin Biomech. (2019) 69:205–14. 10.1016/j.clinbiomech.2019.07.031 31382163 PMC6823156

[21] Mansfield A Peters AL Liu BA Maki BE. Effect of a perturbation-based balance training program on compensatory stepping and grasping reactions in older adults: a randomized controlled trial. Phys Ther. (2010) 90:476–91. 10.2522/ptj.20090070 20167644

[22] Handelzalts S Kenner-Furman M Gray G Soroker N Shani G Melzer I. Effects of perturbation-based balance training in subacute persons with stroke: a randomized controlled trial. Neurorehabil Neural Repair. (2019) 33:213–24. 10.1177/1545968319829453 30767613

[23] Nonnekes J de Kam D Geurts AC Weerdesteyn V Bloem BR. Unraveling the mechanisms underlying postural instability in Parkinson's disease using dynamic posturography. Expert Rev Neurother. (2013) 13:1303–8. 10.1586/14737175.2013.839231 24160682

[24] Hermens HJ Freriks B Disselhorst-Klug C Rau G. Development of recommendations for SEMG sensors and sensor placement procedures. J Electromyogr Kinesiol. (2000) 10:361–74. 10.1016/S1050-6411(00)00027-4 11018445

[25] Kelly VE Bastian AJ. Antiparkinson medications improve agonist activation but not antagonist inhibition during sequential reaching movements. Mov Disord. (2005) 20:694–704. 10.1002/mds.20386 15719427

[26] Lang KC Hackney ME Ting LH McKay JL. Antagonist muscle activity during reactive balance responses is elevated in Parkinson's disease and in balance impairment. PLoS ONE. (2019) 14:e0211137. 10.1371/journal.pone.0211137 30682098 PMC6347183

[27] Nevisipour M Grabiner MD Honeycutt CF. A single session of trip-specific training modifies trunk control following treadmill induced balance perturbations in stroke survivors. Gait Posture. (2019) 70:222–8. 10.1016/j.gaitpost.2019.03.002 30904789 PMC6508877

[28] Crenshaw JR Rosenblatt NJ Hurt CP Grabiner MD. The discriminant capabilities of stability measures, trunk kinematics, and step kinematics in classifying successful and failed compensatory stepping responses by young adults. J Biomech. (2012) 45:129–33. 10.1016/j.jbiomech.2011.09.022 22018682

[29] Grabiner MD Donovan S Bareither ML Marone JR Hamstra-Wright K Gatts S. Trunk kinematics and fall risk of older adults: translating biomechanical results to the clinic. J Electromyogr Kinesiol. (2008) 18:197–204. 10.1016/j.jelekin.2007.06.009 17826181

[30] Kannan L Vora J Varas-Diaz G Bhatt T Hughes S. Does exercise-based conventional training improve reactive balance control among people with chronic stroke? Brain Sci. (2020) 11:2. 10.3390/brainsci11010002 33374957 PMC7821930

[31] Gray VL Juren LM Ivanova TD Garland SJ. Retraining postural responses with exercises emphasizing speed poststroke. Phys Ther. (2012) 92:924–34. 10.2522/ptj.20110432 22421735

[32] Junata M Cheng KC Man HS Lai CW Soo YO Tong RK. Kinect-based rapid movement training to improve balance recovery for stroke fall prevention: a randomized controlled trial. J Neuroeng Rehabil. (2021) 18:150. 10.1186/s12984-021-00922-3 34635141 PMC8503723

[33] Marigold DS Eng JJ Dawson AS Inglis JT Harris JE Gylfadottir S. Exercise leads to faster postural reflexes, improved balance and mobility, and fewer falls in older persons with chronic stroke. J Am Geriatr Soc. (2005) 53:416–23. 10.1111/j.1532-5415.2005.53158.x 15743283 PMC3226796

[34] Pigman J Reisman DS Pohlig RT Jeka JJ Wright TR Conner BC. Posterior fall-recovery training applied to individuals with chronic stroke: a single-group intervention study. Clin Biomech. (2021) 82:105249. 10.1016/j.clinbiomech.2020.105249 33421756 PMC7940569

[35] Buurke JH Nene AV Kwakkel G Erren-Wolters V Ijzerman MJ Hermens HJ. Recovery of gait after stroke: what changes? Neurorehabil Neural Repair. (2008) 22:676–83. 10.1177/1545968308317972 18971383

[36] Den Otter AR Geurts AC Mulder T Duysens J. Gait recovery is not associated with changes in the temporal patterning of muscle activity during treadmill walking in patients with post-stroke hemiparesis. Clin Neurophysiol. (2006) 117:4–15. 10.1016/j.clinph.2005.08.014 16337186

[37] Buma F Kwakkel G Ramsey N. Understanding upper limb recovery after stroke. Restor Neurol Neurosci. (2013) 31:707–22. 10.3233/RNN-130332 23963341

[38] Winters C Kwakkel G van Wegen EEH Nijland RHM Veerbeek JM Meskers CGM. Moving stroke rehabilitation forward: The need to change research. NeuroRehabilitation. (2018) 43:19–30. 10.3233/NRE-172393 30056434

[39] Perera CK Gopalai AA Ahmad SA Gouwanda D. Muscles affecting minimum toe clearance. Front Public Health. (2021) 9:612064. 10.3389/fpubh.2021.612064 34136448 PMC8200481

